# Anti-cancer Substances and Safety of Lactic Acid Bacteria in Clinical Treatment

**DOI:** 10.3389/fmicb.2021.722052

**Published:** 2021-10-12

**Authors:** Chaoran Liu, Jiaqi Zheng, Xuan Ou, Yuzhu Han

**Affiliations:** ^1^College of Animal Science and Technology, Southwest University, Chongqing, China; ^2^Immunology Research Center, Medical Research Institute, Southwest University, Chongqing, China

**Keywords:** lactic acid bacteria, anti-cancer, substances, mechanisms, safety

## Abstract

Lactic acid bacteria (LAB) are a kind of Gram-positive bacteria which can colonize in the biological gastrointestinal tract and play a variety of probiotic roles. LAB have a wide range of applications in industry, animal husbandry, planting, food safety, and medical science fields. Previous studies on LAB have typically concentrated on their effects on improving the digestion and absorption of the gastrointestinal tract, regulating the balance of the microflora, and inhibiting the production and accumulation of toxic substances. The resistance of LAB to cancer is a topic of growing interest and relevance. This paper provided a summary of bio-active substances of LAB when they act against cancer, as well as the safety of LAB in clinical cancer treatment. Moreover, this paper further discussed several possible directions for future research and the potential application of LAB as anti-cancer therapy.

## Introduction

Cancer is a malignant tumor which is caused by gene mutation in normal cells (Visvader, 2011; Rycaj and Tang, 2015). Currently, cancer is the leading cause of death worldwide and a gigantic obstacle to prolonging human life. According to data by the International Agency for Research on Cancer, there were 19.3 million new cancer cases and nearly 10 million fatal cases worldwide in 2020 (Sung et al., 2021). By 2040, the global cancer burden will be heavier, as it is expected to reach 28.4 million cancer cases, a 47% rise from 2020. Therefore, the situations of cancer control and treatment are very severe.

The traditional cancer treatments are mainly surgical resection, radiotherapy, and drug treatment (Qin, 2015). However, radiotherapy could inevitably damage the normal cells around the cancer tissues, causing the breakage of DNA double-strand and destroying the genetic information (Lobrich and Kiefer, 2006), as well as causing progressive fibrosis of blood vessels and soft tissues and reducing the self-healing ability of the body (Yu et al., 2020). Drug therapy can result in damage to normal tissues and organs such as bone marrow, kidney, and oral mucosa and can hinder the normal metabolism. In addition, these three methods may cause inflammation and secondary lymphedema during treatment (Dennert and Horneber, 2006).

## Anti-Cancer Potential of Lab

At present, researchers are constantly exploring cancer therapies with few side effects, and the anti-cancer characteristics of lactic acid bacteria (LAB) are one of the research interests. Lactic acid bacteria (Norouzi et al., 2018), as the dominant probiotics in the intestine, are mostly colonized in part from the duodenum to the end of the ileum (Meng et al., 2020). LAB and their metabolites could enhance immunity, improve gastrointestinal function, increase resistance to obesity, and increase antioxidant abilities, as well as reduce blood glucose concentration and cholesterol (Nowak et al., 2019; Mathur et al., 2020; Wang et al., 2020). In addition, a number of studies have reported that LAB also have an effect on anti-cancer (Mital and Garg, 1995; Nowak et al., 2019; Table 1).

**Table 1:** Lactic acid bacteria with potential anti-cancer roles.

Strain	Cancer cell lines	Background	Anti-cancer mechanisms	References
Six lactic acid bacteria mixture^a^	Chronic myelogenous leukemia K562 cells Colorectal tumor HCT116 cells	*In vitro*	Activate human natural killer KHYG-1 cells	Yamane et al., 2018
*Lactococcus lactis* subsp. *lactis*	Colorectal cancer LS180, SW48, HT29 and Caco2 cells	*In vitro*	Secrete nisin to cause the down-regulation of CEA, CEAM6, MMP2F, MMP9F genes	Norouzi et al., 2018
*Lactobacillus reuteri* ATCC-PTA-6475	Breast cancer cells	*In vivo* (inbreeding Swiss mice)	Trigger CD4+ and CD25+ lymphocytes Block NFκ-B-p65 nuclear translocation and c-jun expression in mammary tumor cells	Lakritz et al., 2014
*Lactobacillus acidophilus* LA1	Ehrlich ascites carcinoma cells	*In vivo* (male albino mice)	Suppress LDH and ALP enzymes	Abd El Ghany et al., 2015
*L. acidophilus*	/	*In vivo* (The patients diagnosed with gastritis or dyspepsia and carried with *Helicobacter pylori*)	Inhibit the growth of *H. pylori* to inhibit the formation of gastric cancer	Du et al., 2012
*Lactobacillus plantarum* NCU116	Intestinal epithelial cancer CT26 cells	*In vitro*	Induce the apoptosis of CT26 cells *via* TLR2 and c-Jun dependent Fas/Fasl-mediated apoptotic pathway	Zhou et al., 2017
*L. acidophilus* 606	Colon cancer HT-29 cells	*In vitro*	Induce Beclin-1 and GRP78 and affect Bcl-2 and Bak regulation	Kim et al., 2010
*L. acidophilus* DM9811	Colon cancer HT-29 cells Ascites hepatoma cells	*In vivo* and *in vitro*	Prolong G1 phase of tumor cells and increase the expression of tumor suppressor gene p53	Zhang et al., 2011
*L. lactis* ATCC 19435	Human breast adenocarcinoma MCF-7 cells Liver hepatocellular carcinoma HepG2 cells	*In vitro*	Secrete nisin to cause the cell shrinkage, cytoplasmic vacuolization, nuclear condensation and lateralization	Paiva et al., 2012
*Lactobacillus casei* Shirota	Bladder cancer cells Colorectal cancer cells	*In vivo*	Enhance the cytotoxicity of NK cells to delay tumor onset	Takagi et al., 2001
*Bififidobacteriuminfantis* ATCC 15697	Meth A fibrosarcoma	*In vivo* (BALB/c mice) and *in vitro*	Inhibit the proliferation of cancer cells by cell wall preparations	Sekine et al., 1985
*L. lactis* subsp. *lactis*	Bladder cancer HT-1376 cells Colon cancer DLD-1 and SNUC2A cells Kidney cancer A498 cells	*In vitro*	Inhibit the proliferation of cancer by peptidoglycan	Kim et al., 2002
*Bifidobacterium adolescentis* *Bifidobacterium breve*	Bladder cancer HT-1376 cells Colon cancer DLD-1 and SNUC2A cells Kidney cancer A498 cells	*In vitro*	Inhibit the proliferation of cancer by peptidoglycan	Kim et al., 2002
*Lactobacillus rhamnosus* GG	Pancreatic ductal adenocarcinoma	*In vivo*	Promote the secretion of Th1-related cytokines IFN and Th2-related cytokines IL-4 in CD4+T cells	Shi et al., 2020
*L. casei* M5 *L. casei* SB27 *L. casei* X12 *L. casei* K11	Colon cancer HT-29 cells	*In vitro*	Induce G0/G1 cell cycle arrest and caspase-3 dependent apoptosis	Di et al., 2018
*Lactobacillus paracasei* subp. *paracasei* X12	Colon cancer HT-29 cells	*In vitro*	Expose the hallmarks of CRT and through the ER-targeted as well as Ca^2+^ signaling pathway to induce immunogenic cell death (ICD)	Tian et al., 2015
*L. casei* ATCC 25180	Various tumour cells	*In vivo*	Trigger the Bax-induced proapoptotic process including a decrease in succinate dehydrogenase activity, cellular ATP and mitochondrial membrane potential, as well as changes in the VDAC structure on the outer mitochondrial membrane and cytochrome C release to induce apoptosis by binding with the mitochondrial-bound hexokinase	Fichera et al., 2016
*L. acidophilus* CICC 6074	Colon cancer HT-29 cells	*In vitro*	Arrest the cell cycle in G1 phase by up-regulating the expression of p53, p21 and p16 and down-regulating the expression of CDK1 (cyclin-dependent kinase) and cyclin B	Zhang et al., 2020
*L. lactis*	Colorectal cancer SW480 cells	*In vitro*	Inhibit cyclin D1 gene expression	Hosseini et al., 2020
*Lactobacillus bulgaricus*	/	*In vivo*	Promote the mitosis of mouse spleen B cells and Pierre spot cells	Kitazawa et al., 2003
*Streptococcus thermophilus*	/	*In vivo*	Promote the mitosis of mouse spleen B cells and Pierre spot cells	Kitazawa et al., 2003

a *Six lactic acid bacteria mixture*:

*L. lactis subsp. lactis*

*L. lactis subsp. cremoris*

*L. lactis subsp. lactis biovar diacetylactis*

*L. plantarum*

*Leuconostoc meseuteroides subsp. cremoris*

*L. casei*

Yamane et al. (2018) reported that the six-LAB (*Lactococcus lactis* subsp. *lactis*, *L. lactis* subsp. *cremoris*, *L. lactis* subsp. *lactis* biovar *diacetylactis*, *Lactobacillus plantarum*, *Leuconostoc meseuteroides* subsp. *cremoris*, and *Lactobacillus casei*) mixture from kefir has strong effects on natural immunity and tumor cell cytotoxicity. In KHYG-1 cells treated with the mixture of six LAB from kefir, mRNA expression and IFN-gamma (interferon gamma) secretion levels were increased which enhanced the cytotoxicity to human chronic myelogenous leukemia K562 cells and colorectal tumor HCT116 cells (Yamane et al., 2018). Lakritz et al. (2014) found that inbreeding Swiss mice could reduce the risk of breast cancer after drinking water containing *Lactobacillus reuteri* ATCC-PTA-6475. In outbred Swiss mice which were fed some westernized chow to increase the risk of mammary tumors, microbially-triggering CD4+, CD25+ lymphocytes may be the anti-cancer mechanism. In genetically susceptible mice, they found that *L. reuteri* ATCC-PTA-6475 blocked NFκ-B-p65 nuclear translocation and c-jun expression in mammary tumor cells to inhibit their growth (Lakritz et al., 2014). LAB could also reduce the risk of cancer by inhibiting the production of pathogens. Du et al. (2012) reported that 234 patients with *Helicobacter pylori*-positive gastritis treated with *Lactobacillus* before or after the standard triple therapy, the C13 or C14 urease breath tests were negative after 4weeks of continuous treatment. This means that *H. pylori* were eradicated, *H. pylori* are the common pathogenic bacteria, which have a certain relationship with the formation of gastric cancer. Therefore, the possibility of gastritis developing into gastric cancer was reduced by this therapy (Du et al., 2012). Moreover, researchers have shown that LAB could degrade the toxins which have a link with cancer to indirectly inhibit cancer development. Guimaraes et al. (2018) found that the supernatant of *L. plantarum* UM55 could inhibit the growth of *Aspergillus flavus* and the production of aflatoxins. The analysis of the photodiode array detector showed that the active substances are organic acids such as phenyllactic acid (PLA), hydroxyphenyllactic acid (OH-PLA), lactic acid, and indole lactic acid (ILA). These organic acids showed strong indication that about 0.87mg/ml PLA, 1.47mg/ml ILA, 1.80mg/ml OH-PLA or 3.92mg/ml lactic acid is sufficient to inhibit the production of 90% aflatoxins (Guimaraes et al., 2018). Aflatoxins are highly toxic. In 1993, aflatoxins were designated as a kind of natural carcinogen by the WHO Cancer Research Institute, which are extremely harmful to humans and animals (Girona et al., 2020). Therefore, organic acids from *L. plantarum* UM55 can reduce the risk of cancer through the removal of aflatoxins.

To some extent, the anti-cancer effects of LAB have promoted the progress of cancer research and provided new ideas for cancer therapy.

## Anti-Cancer Substances of Lab

Many studies have shown that LAB have the function of inhibiting cancer cells, and the active substances and their mechanisms that exert anti-cancer effects are different. Active substances are the basis of LAB anti-cancer properties. Based on recent studies, active substances can be mainly classified into extracellular polysaccharides (EPS), peptidoglycan, nucleic acid, bacteriocin, and S-layer protein five categories.

### Extracellular Polysaccharides

**Figure 1:**
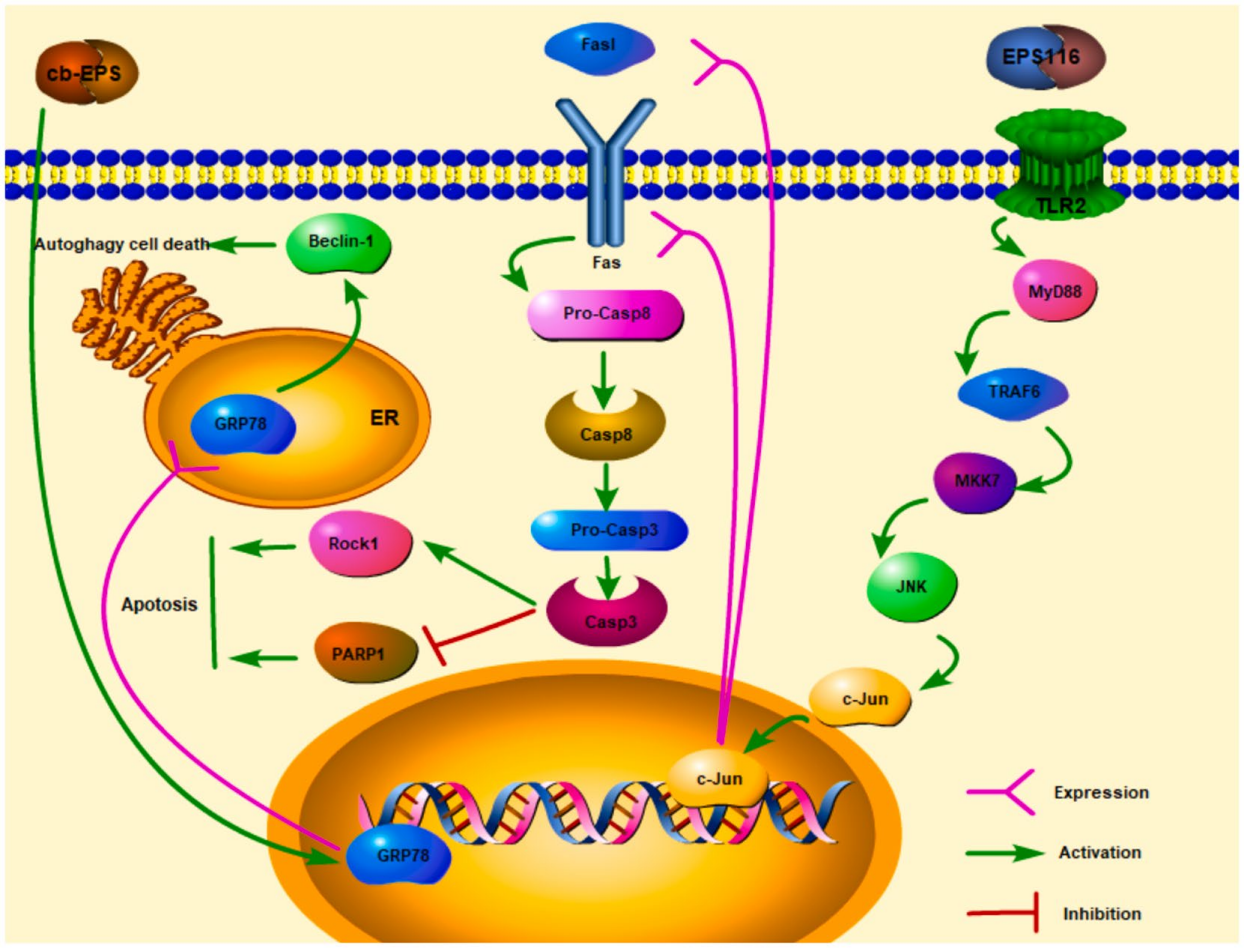
Extracellular polysaccharides (EPS) of lactic acid bacteria kills cancer cells *via* apoptosis and autophagy (
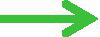
 activation; 
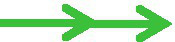
 expression).

Extracellular polysaccharides are one of the polysaccharides which are secreted outside cell walls (Cerning, 1990). In recent years, with the in-depth study of EPS, researchers have explored many physiological functions of EPS, including anti-cancer effects. After EPS from *Lactobacillus acidophilus* LA1 (EPS LA1) treated the male albino mice injected with EAC ascites tumor cells, the solid tumor diameter of the mice can be significantly reduced. On the other hand, there was a marked repression of lactate dehydrogenase (LDH) and alkaline phosphatase (ALP) enzymes (Abd El Ghany et al., 2015). Researchers have discovered a link between LDH, ALP, and tumor. LDH and ALP were related to tumor size, distant metastasis, and cancer recurrence. Inhibition of LDH can cut off the process of glucose conversion to lactate (Glycolytic pathway), so that tumor cells cannot maintain high ATP levels and tumor cells growth may be limited (Ji et al., 2016; Varma et al., 2021). Therefore, EPS LA1 may suppress LDH and ALP to inhibit ascites tumor cells. Zhou et al. (2017) studies showed that EPS116 from *L. plantarum* NCU116 may inhibit the proliferation of colorectal cancer cells CT26 through a cell type manner which significantly suppressed the growth and survival of CT26 in the induction of apoptosis. The mechanism of action was that EPS116 first bound to TLR2 and activated the TLR2/MyD88/TRAF6/MKK7 pathway, which then induced the activation of JNK/c-Jun, and activated c-Jun to upregulate the transcription and translation of Fas and Fasl, leading to Fas-mediated apoptosis signaling pathways. Fas/Fasl signaling is involved in FADD activation of caspase-8 and caspase-3. Activated Caspase-3 facilitated apoptosis by upregulating the expression of cellular target proteins PARPs and Rock1 then enhancing the cleavage of PARP1, so that EPS116 could inhibit the growth of CT26 (Figure 1; Zhou et al., 2017). cb-EPS from *L. acidophilus* 606 extracted from human feces can suppress colon cancer cells HT-29, besides HT-29 cells are significantly inhibited by EPS in a dose and time-dependent manner (Kim et al., 2010). Kim et al. (2010) also discovered that cb-EPS may induce ER stress and GRP78 (ER molecular chaperone) expression, which then promoted autophagy-related cancer cells death through a cascade affected by Beclin-1 (the mammalian autophagy protein) expression (Figure 1). By studying the EPS from *L. casei* M5, *L. casei* SB27, *L. casei* X12, and *L. casei* K11, especially the acidic EPS produced by *L. casei* SB27, EPS exerted the inhibitory effects on HT-29 cells by inducing G0/G1 cell cycle arrest and caspase-3 dependent apoptosis (Di et al., 2018).

Interestingly, the polysaccharide biological functions could have been increased after chemical modification, and now it has been shown that the anti-cancer abilities of polysaccharides are significantly enhanced after modification. Xu et al. (2019) showed that the negative charged phosphate groups on phosphorylated polysaccharides had a high affinity with receptors on the immune cells surface, which effectively activated the immune response and blocked the progress of cancer. Sulfated polysaccharides could also inhibit tumor cells growth by arresting the cell cycle progress in specific phases or activating immune cells (such as macrophages) to improve their ability of recognition and reduction of tumor cells (Xu et al., 2019). Human breast cancer MCF-7 cells and mouse melanoma B16 cells which are treated with phosphorylated polysaccharides are more significantly suppressed in a dose-dependent manner compared with polysaccharides (Deng et al., 2015). EPS after chemical modification can increase the inhibitory effects on cancer cells, and this result may provide new ideas for the research and development of anti-cancer drugs.

### Peptidoglycan

Peptidoglycan known as murein, is an important component of bacterial cell walls. As the protein scaffold of cell walls, peptidoglycan can maintain the normal morphology of cells (Dramsi et al., 2008). Some researchers also found that peptidoglycan has anti-cancer effects by studying the physiological functions of peptidoglycan. Research by Sekine et al. (1985) is the first evidence of the anti-cancer effects of peptidoglycan from *Bififidobacterium infantis* ATCC 15697 by researching Meth A fibrosarcoma in BALB/c mice (Sekine et al., 1985). Matsumoto et al. (2009) showed that the cell wall-derived polysaccharide-peptidoglycan complex (PSPG) in *L. casei* Shirota could improve ileitis and inhibit the activation of IL-6/STAT3 signaling, so as to play a suppressive effect on ileal cancer (Matsumoto et al., 2009). In a similar spirit, Kim et al. (2002) also confirmed that peptidoglycan from *Lactococcus* and *Bifidobacterium* cell walls can inhibit the proliferation of bladder cancer HT-1376, colon cancer DLD-1 and SNUC2A cells, as well as kidney cancer A498 cells (Kim et al., 2002). Peptidoglycan from *Lactobacillus paracasei* subp. *paracasei* X12 could expose the hallmarks of CRT and through the ER-targeted as well as Ca^2+^ signaling pathway to induce immunogenic cell death (ICD) in HT-29 cells (Tian et al., 2015). Fichera et al. (2016) found that peptidoglycan from *L. casei* ATCC 25180 could trigger the Bax-induced proapoptotic process including a decrease in succinate dehydrogenase activity, cellular ATP and mitochondrial membrane potential, as well as changes in the VDAC structure on the outer mitochondrial membrane and cytochrome C release to induce apoptosis by binding with the mitochondrial-bound hexokinase (Fichera et al., 2016).

### Nucleic Acid

There is evidence showing that nucleic acids in the fermentation broth of LAB have anti-cancer effects. The RNA extracted from the logarithmic growth phase medium filtrate of *Lactobacillus* DM9811 are confirmed to have certain inhibitory effects upon colon cancer HT-29 cells and mouse ascites hepatoma cells by MTT method, as well as the inhibitory effects being dose-dependent. RNA can increase the activity of NK cells and CD4+ T cells to upregulate the level of cellular immunity and inhibit cancer cell growth (Zhang et al., 2011). At present, dendritic cells and antigen presenting cells (such as macrophages) can be strongly stimulated by CpG and AT oligodeoxynucleotides, CpG and AT can recognize and bind to TLR9 of the Toll-like receptor family, thereby inducing Th1 immune response, up-regulating the level of anti-cancer immune response, and inhibiting the development of cancer (Bohle et al., 1999; Shimosato et al., 2005). This implies that CpG and AT oligonucleotides have the potential for Th1-like vaccine adjuvants and development as DNA-based cancer vaccines (Krieg, 2000). Studies have shown that DNA fragments of *Lactobacillus bulgaricus* and *Streptococcus thermophilus* can promote the mitosis of mouse spleen B cells and Pierre spot cells, which would enhance the immune functions to improve anti-cancer capacity (Kitazawa et al., 2003).

### Bacteriocin

Lactic acid bacteria produce not only a variety of active substances such as organic acids and reductases during fermentation, but they can also produce bacteriocins with anti-bacterial activity. So far, there has been some initial progress in research with regards to LAB bacteriocins in food preservation (Holzapfel et al., 1995), anti-bacterial, anti-viral (Farías et al., 1996; Twomey et al., 2002), and other fields. At the same time, there is a growing body of research examining the anti-cancer effects of LAB. Respectively, Paiva et al. (2012) studied the cytotoxic effect on MCF-7 cells as well as HepG2 cells of nisin as the typical representative of bacteriocins. The IC50 (concentration at which half of the cells are inhibited) values of 105.46 and 112.25μm are obtained for these two cell lines by MTT colorimetric assay. Meanwhile, the cancer cells shrinkage, cytoplasmic vacuolization, nuclear condensation, and lateralization could be observed under inverted microscope, and finally the cells fall off (Paiva et al., 2012). In addition, Maher and McClean (2006) found that LDH in these two treated cell lines are significantly increased, indicating that nisin could destroy the integrity of the plasma membrane of cell lines to cause the death of cancer cells (Maher and McClean, 2006). The effects of different doses of nisin A generated from *L. lactis* subsp. *lactis* on human colon adenocarcinoma cell lines LS180, SW48, HT29, and Caco-2 are reported by Norouzi et al. (2018). The final data showed that the concentration of 40–50IU/ml of nisin A could inhibit the proliferation of LS180, 250–350IU/ml of nisin A could inhibit the division of SW48, HT29, and Caco-2 cells, as well as the research also showing that nisin A could inhibit the proliferation of cancer cells by down-regulating the gene expression of CEA, CEAM6, and MMP2 F, which have been associated with tumor progression, invasion, and metastasis (Norouzi et al., 2018). Hosseini et al. (2020) have discovered that the expression level of cyclin D1 gene is decreased on SW480 cancer cell line after treating nisin from *Lactococcus lactis*. This is owing to D-type cyclins playing a key role in the cell cycle, and over-expression of cyclin D is associated with tumor proliferation. Therefore, nisin from *L. lactis* could inhibit SW480 cancer cell line by inhibition of cyclin D1 gene expression (Hosseini et al., 2020). Prince et al. (2019) explored the mechanism by which nisin inhibited neuroblastoma cells by modulation of phase behavior and cell membrane fluidity. Nisin interaction with a neuroblastoma cancer cell resulted in enhancing membrane fluidity and reduction in the dipole potential, which inhibited neuroblastoma cell growth (Prince et al., 2019).

### S-layer Protein

**Figure 2:**
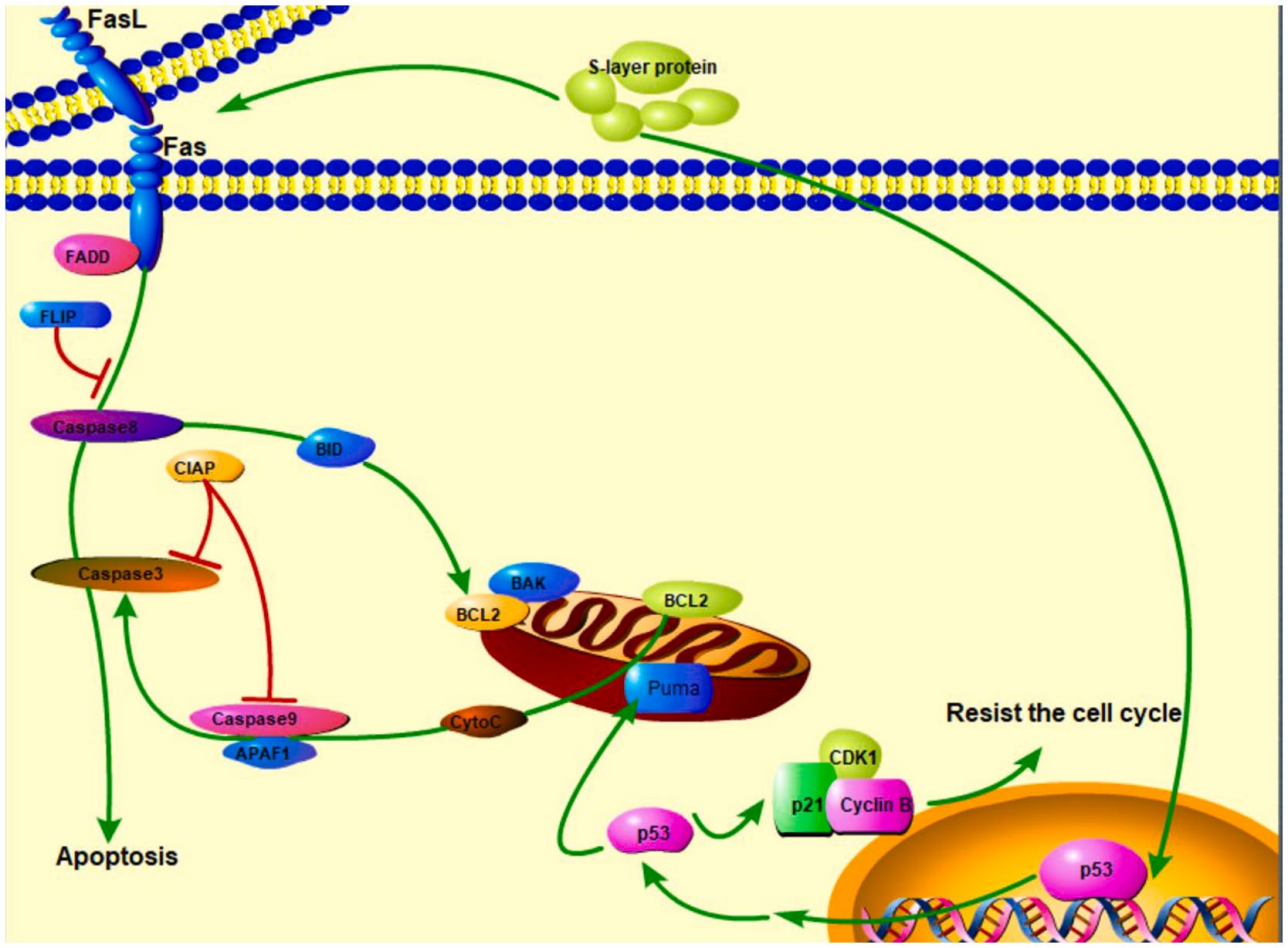
S-layer proteins of *L. acidophilus* kill cancer cells *via* apoptosis and resistance of cell cycle (
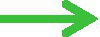
 activation; 
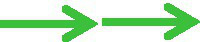
 expression; 
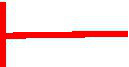
 inhibition).

The surface layers, referred as S-layers, are the two-dimensional crystalline arrays of cell surface proteins or glycoprotein subunits (Zhu et al., 2017). S-layer proteins in LAB are not only cell surface structures used for aggregation, but also play an important role in intestinal tissue adhesion together with some other functional elements (Alp et al., 2020). On the other hand, the researchers have found that S-layer protein has a role in cancer suppression. Zhang et al. (2020) proposed that S-layer protein may induce HT-29 cell apoptosis through the death receptor apoptotic pathway and mitochondrial pathway. *L. acidophilus* CICC 6074 S-layer proteins exert their cytotoxic activity against colon cancer HT-29 cells by arresting the cell cycle in G1 phase by up-regulating the expression of p53, p21, and p16 and down-regulating the expression of CDK1 (cyclin-dependent kinase) and cyclin B (Figure 2; Zhang et al., 2020). Wu et al. (2019) reported that the S-layers localized on the surface of S-CM-HPAD NPs and potentiated the immune response to the antigen. It was based on the surface (S-layer) protein-enhanced immunotherapy strategy of cell membrane-coated S-CM-HPAD nanoparticles, which effectively triggered protective immunity of tumors on the construction of melanoma models, confirming the important role of S-layer proteins in inhibiting the growth and metastasis of malignant tumors *via* immunity (Wu et al., 2019).

## Safety of Lab Clinical Treatment

Although a large number of experimental data have shown that LAB have anti-cancer effects, their safety needs to be deeply explored in order to use LAB for clinical treatment. This review elaborates on the safety of LAB from three aspects: LAB have antibiotic resistance; potential toxicity of some enzymes in LAB; degradation of mucin by LAB.

### LAB Have Antibiotic Resistance

Antibiotics are commonly used in the clinical treatment of diseases caused by microorganisms (Ahmed et al., 2020). Antibiotics have been used in the treatment of diseases for nearly a hundred years. Due to the extensive application of antibiotics, organisms are resistant to most antibiotics. The emergence of antibiotic resistance may lead to ineffective treatments of multiple diseases and cause human death. Therefore, it is of practical significance to study the sensitivity of LAB to antibiotics. Coppola et al. (2005) measured the susceptibility of 63 *Lactobacillus rhamnosus* strains, *Lactobacillus* GG, and the type strain *L. rhamnosus* DSM 20021 from Parmigiano Reggiano cheese to 41 antibiotics (Coppola et al., 2005). By using the disk diffusion method and measuring the diameter of inhibition zone, the results were that all strains isolated from cheese were resistant to six antibiotics (cefixime, vancomycin, neomycin, enoxacin, pefloxacin, and sulphamethoxazole plus trimethoprim), *L. rhamnosus* DSM20021 was resistant to 9 antibiotics (the previous 6 plus cephalosporins, bacitracin, and lincomycin), and *L*.GG was resistant to 18 antibiotics. Researchers reported the susceptibility of 141 lactobacilli strains from natural whey starter cultures, ripened Grana Padano and Parmigiano Reggiano cheeses to 13 antibiotics. And their results showed that the strains from Parmigiano Reggiano were more resistant to gentamicin and penicillin G. On the other hand, the strains isolated in the ripened cheese were generally more resistant than those isolated from natural whey starter cultures (Belletti et al., 2009). *L. plantarum* isolated from soft cheese by Teuber et al. (1999) are resistant to tetracycline (Teuber et al., 1999). Charteris et al. (1998) described the resistance spectrum of LAB more than 20years ago. The results showed that LAB are resistant to ampicillin, penicillin G, cephalosporin, bacitracin, chloramphenicol, erythromycin, clindamycin, nitrofurantoin, tetracycline, resistant to vancomycin, gentamicin, kanamycin, streptomycin, fusidic acid, and so on (Charteris et al., 1998; Table 2). Researchers have found that the antibiotic resistance gene of LAB could be transferred so that the pathogens developed antibiotic resistance. Researchers have found that antibiotic resistance genes could transfer resistance genes to pathogens through mobile genetic elements such as plasmids, transposons, insertion sequences, and introns and through conjugation pilus, allowing pathogens to acquire antibiotic resistance (Ojha et al., 2021). Conjugation, which is a type of lateral gene transfer, may make pathogens resistant to antibiotics and thus they cannot be eradicated. The drug resistance of LAB has dual effects. On the one hand, it is conducive to the survival of the bacteria, on the other hand, if the resistance genes of LAB are transferred to pathogens, it would be a great threat to human survival.

**Table 2:** Lactic acid bacteria with antibiotic resistance.

*Bifidobacterium*		*Lactobacillus* (Dec et al., 2015)									
(Charteris et al., 1998)		*L. plantarum*		*L. johnsonii*		*L. ingluviei*		*L. salivarius*		*L. reuteri*	
Resistant	Moderately resistant	Resistant	Moderately resistant	Resistant	Moderately resistant	Resistant	Moderately resistant	Resistant	Moderately resistant	Resistant	Moderately resistant
Cefoxitin	Ceftazidime	Tetracycline	Neomycin	Tetracycline	Amoxicillin	Tetracycline	Doxycycline	Tetracycline	Tylosin	Enrofloxacin	Tetracycline
Aztreonam	Netilmicin	Doxycycline	Tylosin	Doxycycline	Ampicillin	Neomycin	Lincomycin	Neomycin	Doxycycline	Flumequine	Doxycycline
Vancomycin	Sulphamethoxazole	Lincomycin	Ampicillin	Neomycin		Enrofloxacin		Lincomycin	Amoxicillin	Lincomycin	Neomycin
Amikacin	Cotrimoxazole	Flumequine		Lincomycin		Flumequine		Enrofloxacin		Tylosin	Ampicillin
Gentamicin	Ciproflfloxacin	Enrofloxacin		Tylosin				Flumequine			
Kanamycin				Enrofloxacin							
Streptomycin				Flumequine							
Fusidic acid											
Trimethoprim											
Norflfloxacin											
Nalidixic acid											
Metronidazole											
Polymyxin B											
Colistinsulphate											

### Potential Toxicity of Some Enzymes in LAB

Enzymes produced by LAB are closely related to maintaining the survival of bacteria and promoting the probiotic functions of LAB. However, in recent years, some studies have shown that some enzymes produced by LAB can catalyze adversely metabolic activities and threaten the health of humans. Bernardeau et al. (2006) summarized the enzymes that may adversely induce metabolic activities, such as azo reductase, nitroreductase, β-glucuronidase, glycosidase, amino acid decarboxylase, and so on. They may cause hyaluronic acid degradation, platelet aggregation thrombosis, toxic metabolites (such as biogenic amines) and so on (Bernardeau et al., 2006). Some lactobacilli species also have bile salt deconjugase activity. Bile salt deconjugase enzymes can cause some destruction of gastrointestinal digestion. If deconjugation occurs in the small intestine, fat digestion will be disrupted. Similarly, if the large intestine could not deconjugase, the enterohepatic circulation is disrupted and the bile pool is reduced, which leads to impaired fat digestion and absorption. Glucosidases can exert toxic effects by cleaving compounds such as cysteins (which release methyl methoxymethanol) or cyanogenic glycosides (which release hydrogen cyanide). β-glucuronidase can hydrolyze glucuronic acid thus lead to the return of toxicants removed by the liver to the circulation (Bernardeau et al., 2006).

At present, some researchers have found biogenic amines in fermented sausages, which mean that amino acid decarboxylase in LAB may promote amino acid decarboxylation to form biogenic amines (Bover-Cid et al., 2001). Biogenic amines have important impacts on humans. Appropriate amounts of biogenic amines would stabilize blood pressure, transmit information as potential neurotransmitters, regulate the synthesis of DNA, RNA, and proteins, as well as maintain the stability of biofilm. A large number of biogenic amines can cause food poisoning, generate precursors of carcinogens and nitroso compounds. Like cadaverine and putrescine could inhibit the activity of histamine and tyramine metabolic enzymes, increase the number of histamine and tyramine, fully cause digestive disorders and abnormal blood pressure, and even cause neurotoxicity. In addition, putrescine and cadaverine can react with nitrite to produce carcinogenic substances. Some researchers reported the existence of nitrate reductase in LAB. LAB isolated from Spanish dry-cured sausage are taken as the research object by Landeta et al., and it was found that all strains have nitrate reductase activity (Landeta et al., 2013). Nitrate reductase, as a kind of oxidoreductase, can convert nitrates into nitrites. Nitrites are the precursor of nitrosamines, which is a kind of strong carcinogen. It is very likely to cause cancer and pose a great threat to human health. However, some LAB also can produce nitrite reductase and have the capability to reduce nitrite (Paik and Lee, 2014). Paik and Lee (2014) showed that four tested LAB strains (*L. brevis* KGR3111, *L. curvatus* KGR 2103, *L. plantarum* KGR 5105 and *L. sakei* KGR 4108) isolated from kimchi had the capability to produce nitrite reductase, which apparently reduced nitrite level (Paik and Lee, 2014).

From the perspective of the biological toxicity of some enzymes, LAB have potential toxicity and may induce adversely metabolic activities. Therefore, the development of LAB as cancer treatment drugs needs reasonable strain screening and clinical trials.

### Degradation of Mucin by LAB

Mucins are a kind of mucopolysaccharides composed by glycoproteins. Mucins are involved in the formation of mucus, which can play a role in tissue lubrication and cell signal. It is also the first barrier for the interaction and diffusion of nutrients and intestinal drugs, so that they can be absorbed and enter the circulatory system. Therefore, the existence of mucins can protect the gastrointestinal tract from the invasion of pathogenic bacteria and toxic metabolites, and provide a relatively suitable environment for the body with less interference factors, thus maintain the homeostasis of various functions and promote the smooth and orderly progress of various metabolic activities. But studies have found that LAB may degrade mucins. Hoskins (1993) have shown that glycosidases and glucosulphateesterases isolated from Bifidobacterium species can degrade the carbohydrate chain of mucins and release them in the form of monosaccharide components to cause mucins deactivated (Hoskins, 1993). This would make pathogens and toxins susceptible to invading organisms to threaten human health. However, some researchers also reported that *L. rhamnosus* HN001, *L. acidophilus* HN017, and *Bifidobacterium lactis* HN019 could not degrade mucins *in vitro*. These three LAB strains were incubated at 37°C for 48h with porcine gastric mucin (HGM, 0.3%) as substrates, and then any decreases in carbohydrate and protein concentrations in the ethanol-precipitated fraction of the medium were measured using phenol-sulfuric acid and bicinchoninic acid (BCA) protein assays, respectively, and changes in the molecular weight of mucin glycoproteins were monitored by SDS-polyacrylamide gel electrophoresis (SDS-PAGE). The result was that no mucin fragments were derived from mucin suspension and no mucolytic zone was found on agarose (Zhou et al., 2001). It can be argued that although LAB may have the potential hazard of degrading mucins which can lead to invasion of pathogenic bacteria and their metabolites, this hazard can be eliminated by the screening of LAB.

The safety of the clinical application of LAB remains to be further confirmed, and conservatively, the development of LAB as cancer therapeutics still requires in-depth studies and clinical trials.

## Future Directions

Although LAB have potential anti-cancer effects, the safety study of LAB is not very clear and needs further in-depth exploration, it could be known that safe and non-toxic anticancer species can be obtained from the screening of strains to be applied for cancer treatment effectively. The researchers reported that *L. johnsonii* has three active bile salt hydrolase (bsh) genes that can persist in the mouse intestine; on the other hand, *L. helveticus* cannot persist because its bsh gene has a frame-shift, resulting in enzyme inactivation. Based on this study, researchers could reduce bsh enzymes by genome modification which makes some LAB bsh gene frames shift, and the modification could maintain homeostasis of the intestinal environment (Plavec and Berlec, 2020). Therefore, genome modification of LAB can lead to the inactivation of some cellular functions. It can be deleted or mutated by the corresponding gene to reduce harmful function. Developing LAB and its anti-cancer substances as anti-cancer drugs is a promising approach to solve the side effects of surgery, radiotherapy, and chemotherapy. The success of this research would become a major progress for humans to overcome cancers. Perhaps in the future, cancer would no longer be a complication or a source of fear for human beings but would become just like preventing and controlling flus. Therefore, future studies need to conduct more in-depth studies on the anti-cancer effects of LAB especially at the molecular and genetic levels, and to explore ways to improve the safety of LAB such as strain screening and genetic modification, ousing anti-cancer substances from LAB that are safe, non-toxic, and have good anti-cancer effects with reduced adverse effects.

## Author Contributions

CL proposed the article idea and wrote the manuscript. YH guided, revised, and funded this project. JZ and XO made the figures and participated in literature collection, writing and revision. All authors contributed to the article and approved the submitted version.

## Funding

This work was supported by National Natural Science Foundation of China (31901929) and Fundamental Research Funds for the Central Universities (XDJK2020B015).

## Conflict of Interest

The authors declare that the research was conducted in the absence of any commercial or financial relationships that could be construed as a potential conflict of interest.

## Publisher’s Note

All claims expressed in this article are solely those of the authors and do not necessarily represent those of their affiliated organizations, or those of the publisher, the editors and the reviewers. Any product that may be evaluated in this article, or claim that may be made by its manufacturer, is not guaranteed or endorsed by the publisher.
